# Genome-Wide Identification and Evolutionary Analysis of m6A-Related Gene Family in Poplar Nanlin895

**DOI:** 10.3390/plants14132017

**Published:** 2025-07-01

**Authors:** Zeyu Li, Rongxia Liu, Mingqiang Zhu, Jinye Zhang, Zhoujin Li, Kaixin Huang, Zehua Ren, Yan Zhao, Keming Luo, Qin Song

**Affiliations:** Key Laboratory of Eco-Environments of Three Gorges Reservoir Region, Ministry of Education, Chongqing Key Laboratory of Forest Resource Innovation and Utilization, Integrative Science Center of Germplasm Creation in Western China (Chongqing) Science City, School of Life Sciences, Southwest University, Chongqing 400715, China; lzy20226040@swu.edu.cn (Z.L.); lllrx92@163.com (R.L.);

**Keywords:** m6A, genome-wide analysis, gene family, NL895, Cd stress

## Abstract

**Background:** N6-methyladenosine (m6A) is the most prevalent chemical modification of eukaryotic RNA, playing a crucial role in regulating plant growth and development, stress responses, and other essential biological processes. The enzymes involved in m6A modification—methyltransferases (writers), demethylases (erasers), and recognition proteins (readers)—have been identified in various plant species; however, their roles in the economically significant tree species *Populus deltoides* × *P. euramericana* (NL895) remain underexplored. **Results:** In this study, we identified 39 m6A-related genes in the NL895 genome, comprising 8 writers, 13 erasers, and 18 readers. Evolutionary analysis indicated that the expansion of writers and readers primarily resulted from whole-genome duplication events. Purifying selection pressures were observed on all duplicated gene pairs, suggesting their essential roles in functional differentiation. Phylogenetic analysis revealed that writers, erasers, and readers are categorized into six, four, and two groups, respectively, with these genes being more conserved among dicotyledonous plants. Gene structure, protein domains, and motifs exhibited greater conservation within the same group. Promoter analysis of m6A-related genes showed enrichment of *cis*-acting elements associated with responses to light, phytohormones, and stress, indicating their potential involvement in gene expression regulation. Under cadmium treatment, the expression of all writers was significantly upregulated in both the aboveground and root tissues of NL895. **Conclusions:** This study systematically identified m6A-related gene families in *Populus deltoides* × *P. euramericana* (NL895), elucidating their evolutionary patterns and expression regulation characteristics. These findings provide a theoretical foundation for analyzing the molecular mechanisms of m6A modification in poplar growth, development, and stress adaptation, and offered candidate genes for molecular breeding in forest trees.

## 1. Background

N6-methyladenosine (m6A) is the most prevalent chemical modification of eukaryotic RNA and serves as a conserved post-transcriptional gene expression regulation mechanism [1,2]. This dynamic and reversible modification is installed by m6A methyltransferases (writers) such as METTL3, METTL4, WTAP, VIRILIZER, and HAKAI, and can be removed by m6A demethyltransferases (erasers) containing the ALKBH domain [2,3,4,5,6,7]. m6A modifications are recognized by m6A recognition enzymes (readers) containing the YTH domain, facilitating various functions [8,9]. Consequently, m6A plays a crucial role in RNA metabolism, including splicing, stability, transport, and translation, thereby regulating numerous biological processes [2,10,11]. Recent studies have demonstrated that m6A is involved in processes such as animal embryonic development, cell fate determination, and disease resistance [12,13,14]. However, its roles in plants, particularly in woody species, remain less explored.

N6-methyladenosine (m6A) has emerged as a crucial regulatory mechanism in plants, affecting a wide range of biological processes, including growth, organ development, and stress resistance. Studies in the model plant *Arabidopsis thaliana* have revealed that m6A modification is essential for embryogenesis [3], floral transition [15], leaf morphogenesis [16], and root development [17]. Research in other plants has indicated that m6A plays important roles in regulating sporogenesis [18] and fruit ripening [19,20,21]. Additionally, m6A plays a significant role in plant responses to environmental stresses, such as salinity [22], drought [23], and pathogen attacks [24], by modulating the stability and translation of stress-related transcripts. The dynamic collaboration between m6A writers, erasers, and readers ensures precise spatiotemporal control of gene expression, highlighting m6A as a versatile regulator of plant adaptability. However, studies on the regulation of m6A-related genes in long-lived woody plants are still limited.

Poplar (*Populus deltoides* × *P. euramericana*), particularly the NL895 cultivar, serves as a model for studying tree biology, including wood formation, seasonal dormancy, and long-term stress resistance [25]. This fast-growing species is prevalent in southern China and has been extensively studied due to its economic value and ecological significance [26,27,28,29]. However, the m6A-related gene family in NL895 remains underexplored. Therefore, comparative analyses of m6A writers, erasers and readers are necessary and meaningful for learning the tree-specific biological RNA methylation regulation processes.

In this study, we identified and compared proteins from the three m6A-related gene families in NL895. We conducted analyses on their chromosomal locations, syntenic genes, phylogenetic relationships, gene structures, evolutionary pressures, *cis*-acting elements, and gene expression patterns to explore their evolutionary history. Additionally, we examined the expression of m6A writers in NL895 under cadmium stress to investigate their roles in stress response. Our findings provide a theoretical foundation for analyzing the molecular mechanisms of m6A modification in poplar growth, development, and stress adaptation, and offer candidate genes for molecular breeding in forest trees.

## 2. Materials and Methods

### 2.1. Identification of m6A-Related Members in NL895

To identify candidate members of the m6A regulatory machinery in the NL895 genome, the protein sequences of m6A writers, erasers, and readers reported in *Arabidopsis thaliana* [2] were used as queries for a BLASTP (v2.12.0+) search (E-value < 1 ×10^−5^) against the NL895 genomic protein sequences (available at: https://www.ncbi.nlm.nih.gov/search/all/?term=%20PRJNA1061373, accessed on 1 July 2025) [29], using TBtools-II [30]. The conserved domains of all candidate proteins were confirmed using CDD (v3.21, https://www.ncbi.nlm.nih.gov/Structure/cdd/wrpsb.cgi, accessed on 1 July 2025) [31], Pfam (v37.4, http://pfam.xfam.org/, accessed on 1 July 2025) [32], and SMART (v9, http://smart.embl.de/, accessed on 1 July 2025) [33]. The identified m6A writers, erasers, and readers were subsequently renamed based on homology to m6A-related genes in *A. thaliana* (Table S2). Similarly, m6A-related proteins of *Populus trichocarpa* were detected and renamed using the same method (Table S2). The genomic protein sequences of *P. trichocarpa* were obtained from Phytozome (v13, https://phytozome-next.jgi.doe.gov/info/Ptrichocarpa_v4_1, accessed on 1 July 2025).

### 2.2. Characterization of m6A-Related Proteins in NL895

The ProtParam tool on the ExPASy server (https://www.expasy.org/, accessed on 1 July 2025) [34] was used to predict the physical and chemical properties of m6A-related proteins, including molecular weight (MW), grand average of hydropathicity (GRAVY), instability index, and isoelectric point (pI). The subcellular localization of m6A-related proteins was predicted online using BUSCA (http://busca.biocomp.unibo.it, accessed on 1 July 2025) [35].

### 2.3. Chromosomal Location and Synteny Analysis

The genomic location information for m6A writers, erasers, and readers in NL895 was obtained from the GFF annotation file (https://www.ncbi.nlm.nih.gov/search/all/?term=%20PRJNA1061373, accessed on 1 July 2025). TBtools-II [30] was used to map the locations of m6A-related genes on the chromosomes. MCScanX [36] was employed to identify gene duplication events and perform syntenic analysis.

### 2.4. Phylogenetic Tree Analysis

First, the protein sequences of m6A writers, erasers, and readers from *Arabidopsis*, *Oryza sativa*, *P. trichocarpa*, and *P. deltoides* × *P. euramericana* were aligned using ClustalW in MEGA11 [37]. The alignment results were then used to construct a Neighbor-Joining tree in MEGA11 with 1000 bootstrap repeats. The phylogenetic trees were subsequently visualized using Evolview v2 (https://evolgenius.info//evolview-v2, accessed on 14 February 2025) [38].

### 2.5. Gene Structure and Motif Analysis

Gene structures were visualized using TBtools-II [30], while conserved motifs were predicted using the MEME tool [39].

### 2.6. Cis-Acting Element Identification

*Cis*-acting elements in the 2000 bp upstream sequences of m6A-related genes were predicted using PlantCARE (https://bioinformatics.psb.ugent.be/webtools/plantcare/html/, accessed on 1 July 2025).

### 2.7. Gene Expression Analysis

The RNA-seq data from hormone treatments were obtained from previous studies 2.7 [40]. Clean sequence reads were mapped to the reference genome using HISAT2 [41] with default parameters. Using HTSeq software (v0.9.1), raw read counts were generated [42], and the normalization of gene expression levels in terms of FPKM (fragments per kilobase million) was performed by edgeR [43]. Gene expression profiling was visualized using TBtools-II [30].

### 2.8. Plant Materials, Growth Conditions, Cd Treatments and Phytohormone Treatments

Seedlings of NL895 were grown in a greenhouse under a 16 h day/8 h night cycle at 25 °C with 10,000 lux supplemental light. Two-week-old seedlings were treated with 200 µmol/L CdCl_2_ for 24 h. We sprayed 0.2 µmol/L MeJA or 0.5 µmol/L SA on both sides of the leaves until small water droplets formed. Leaf samples were collected after 0 h, 2 h, 6 h, 12 h and 24 h after the treatment.

### 2.9. Total RNA Extraction and Real-Time qPCR (RT-qPCR)

Total RNA from NL895 was extracted using the Biospin Plant Total RNA Extraction Kit (Bioflux, Hangzhou, China). cDNA synthesis was performed using the PrimeScript^TM^ RT Reagent Kit with gDNA Eraser (TaKaRa, Dalian, China). RT-qPCR was carried out using SYBR Premix ExTaq^TM^ (TaKaRa, Dalian, China) in a qTOWER3G IVD Real-Time PCR machine (Analytik Jena AG, Berlin, Germany). The PtoUBQ gene was used as an internal control, and the ΔΔCt method was employed to calculate RT-qPCR data. The primers are listed in Table S1.

## 3. Results

### 3.1. Identification of m6A-Related Genes

**Figure 1:**
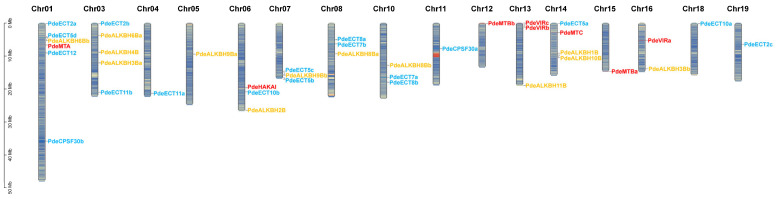
Chromosomal localization of m6A-related genes in NL895. Genes are color-coded: red for writers, yellow for erasers, and sky blue for readers.

To identify members of the m6A-related gene family, we used protein sequences for six writers, thirteen erasers, and thirteen readers from *Arabidopsis thaliana* as query sequences and conducted a BLASTp search against the NL895 genome. After performing a conserved domain screening of the candidate proteins, we identified eight writers (containing MT_A70, Virilizer, HAKAI/CBLL2, or FIP37), thirteen erasers (containing ALKBH), and eighteen readers (containing YTH). All identified members were renamed based on their homology with *A. thaliana* genes, and the details are provided in Table S2. Chromosome localization analysis revealed that writers, erasers, and readers were located on 5, 10, and 11 chromosomes, respectively (Figure 1).

Based on the identification results, we observed an expansion of m6A-related genes in NL895 compared to *Arabidopsis*. To explore the evolutionary relationships of these genes, we analyzed their duplication types (Table S2). Whole-genome duplication (WGD) events played a significant role in the expansion of erasers (6/13) and readers (15/18) in NL895, while dispersed duplication (DSD) events were more frequent in writers (4/8). Only *PdeECT5a* was found to belong to tandem duplication (TD). Neither proximal duplication (PD) nor transposed duplication (TRD) was detected in any of the three gene families, suggesting that m6A-related genes in NL895 may not have been influenced by these types of duplication events. These findings suggest that DSD was the primary driving force for the expansion of writers, while WGD primarily contributed to the expansion of erasers and readers in NL895.

### 3.2. Phylogenetic Analysis of m6A-Related Gene Families

To investigate the phylogenetic and evolutionary relationships among m6A-related genes, we constructed three separate phylogenetic trees using protein sequences from writers, erasers, and readers in four plant species (*A. thaliana*, *Oryza sativa*, *P. trichocarpa*, and NL895). Protein sequences from *A. thaliana* were used as a reference to identify m6A-related proteins in *P. trichocarpa* by the same criteria. The m6A-related protein sequences of *O. sativa* were downloaded from previous studies [2]. *A. thaliana* and *P. trichocarpa* served as dicotyledonous representatives, while *O. sativa* was used as a monocotyledonous outgroup, ensuring the accuracy of the phylogenetic analysis.

**Figure 2:**
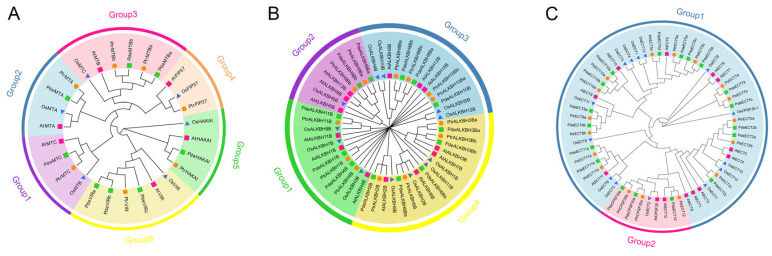
Phylogenetic trees of m6A-related genes from NL895, *A. thaliana*, *O. sativa*, and *P. trichocarpa*. (**A**) Writers; (**B**) erasers; (**C**) readers. The neighbor-joining phylogenetic trees were constructed using MEGA 11 with 1000 bootstrap replicates. Genes from NL895 are represented by lime green rectangles, *A. thaliana* by deep pink rectangles, *O. sativa* by royal blue triangles, and *P. trichocarpa* by dark orange circles.

The phylogenetic trees revealed that writers clustered into six groups: *MTA*, *MTB*, *MTC*, *FIP37*, *HAKAI*, and *VIR* (Figure 2A). Surprisingly, *PdeFIP37* was not detected in NL895. Erasers clustered into four groups, and readers clustered into two groups, *ECT* and *CPSF30* (Figure 2B,C). The *PdeECT* group contained the conserved YTHDF domain, and the *PdeCPSF30* group contained the conserved YTHDC domain. These findings were consistent with previous classifications in plants, indicating that m6A-related genes are highly conserved among plant species.

### 3.3. Gene Structure, Conserved Domain, and Motif Analysis

**Figure 3:**
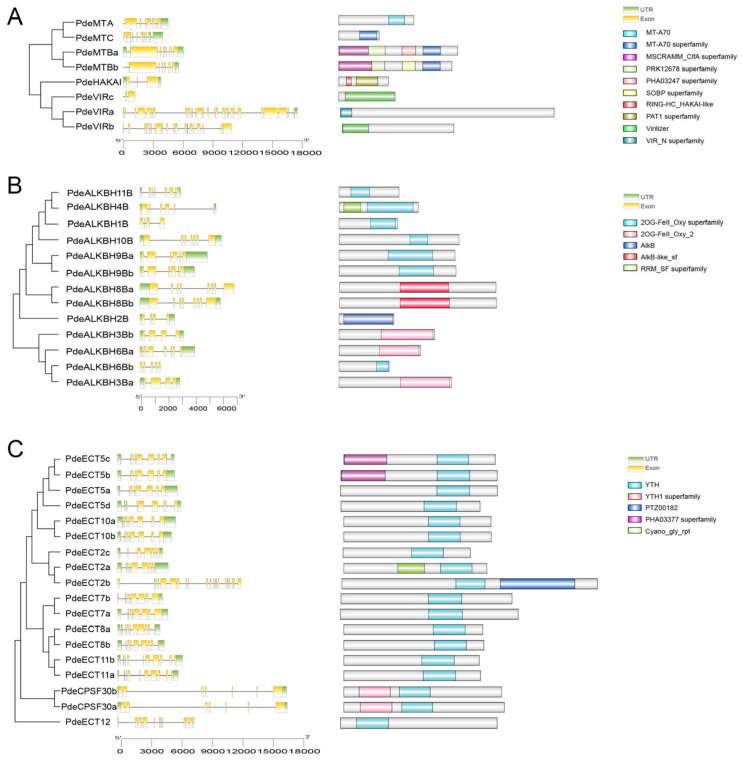
Gene structures and protein domains of m6A-related genes. The neighbor-joining phylogenetic trees, gene structures, and protein domains of writers (**A**), erasers (**B**), and readers (**C**) are shown. Yellow boxes indicate exons, gray lines represent introns, and green boxes highlight untranslated regions (UTRs). Different protein domains are distinguished by colored boxes.

We constructed phylogenetic trees for the three m6A-related gene families using the neighbor-joining method and analyzed the exon–intron structure, conserved domains, and motifs (Figure 3). The m6A-related genes in NL895 exhibited a range of 2 to 27 introns, with the fewest being found in *PdeHAKAI* and *PdeVIRc* (2 introns) and the most in *PdeVIRa* (27 introns). Genes located on the same branch of the evolutionary tree displayed similar exon–intron patterns, suggesting a conserved evolutionary nature among these genes. Conserved domain analysis confirmed that all identified proteins contained specific domains: the MT-A70 domain in writers, the AlkB domain in erasers, and the YTH domain in readers. Conserved motif analysis revealed 16 distinct motifs in the m6A-related genes of NL895 (Figure S1). Notably, genes clustered on the same branch shared similar motifs, although the number and type of motifs varied significantly between groups.

### 3.4. Prediction of Physicochemical Properties and Subcellular Localization of m6A-Related Proteins

The lengths of m6A-related proteins in NL895 ranged from 219 amino acids (PdeALKBH6Bb) to 2187 amino acids (PdeVIRa) (Table S3). The average lengths of writers, erasers, and readers were 959 aa, 423 aa, and 664 aa, respectively. The molecular weights (MWs) of these proteins ranged from 24,274.32 Da to 239,451.46 Da, with average MWs of 105,736.42 Da for writers, 46,896.74 Da for erasers, and 73,119.15 Da for readers. Additionally, 5, 9, and 17 of the writers, erasers, and readers, respectively, were classified as acidic proteins (with isoelectric points < 7). Furthermore, 7, 12, and 11 proteins from writers, erasers, and readers had instability index values greater than 40. All m6A-related proteins in NL895 exhibited negative grand average of hydropathicity (GRAVY) values, indicating that these proteins are hydrophilic. Subcellular localization analysis showed that all writers and most erasers and readers in NL895 were localized in the nucleus (Table S3).

### 3.5. Synteny Analysis of m6A-Related Genes

**Figure 4:**
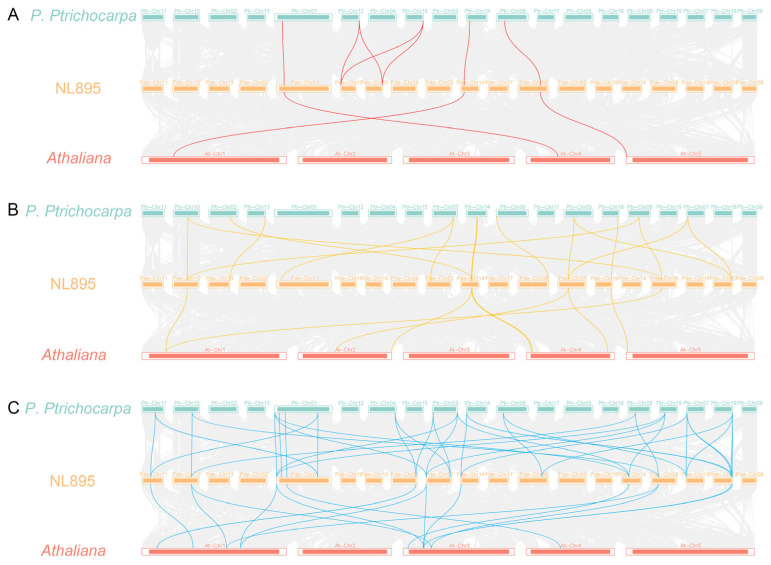
Genome-wide collinearity analysis of m6A-related genes from NL895, *A. thaliana*, and *P. trichocarpa*. (**A**) Writers; (**B**) erasers; (**C**) readers. Gray lines represent collinear blocks between NL895 and the other two plant genomes, while red, yellow, and sky blue lines highlight collinear gene pairs of writers, erasers, and readers, respectively.

Synteny analysis revealed three and seven orthologous gene pairs for writers between NL895 and *Arabidopsis* or *P. trichocarpa*, respectively (Figure 4A). Similarly, 8/16 and 16/37 orthologous pairs were identified for erasers and readers between NL895 and *Arabidopsis* or *P. trichocarpa*, respectively (Figure 4B,C). These results suggest that these gene pairs had been present before the divergence of the three species. *Ka*/*Ks* analysis indicated that the *Ka*/*Ks* ratios of the 12 duplicated gene pairs were less than 1, suggesting that these gene pairs had underwent purifying selection (Table 1).

### 3.6. Cis-Acting Elements Analysis in m6A-Related Genes

**Figure 5:**
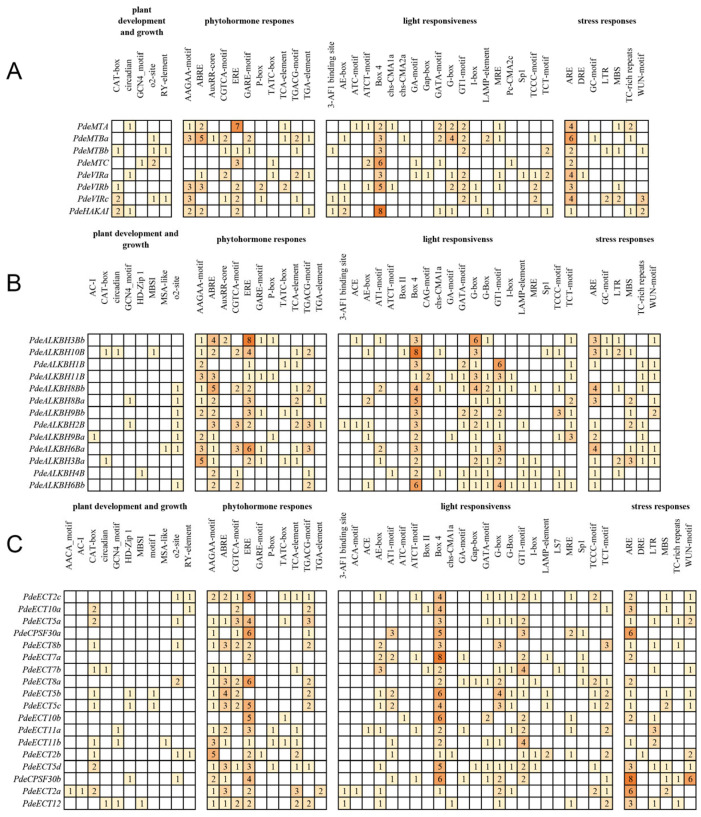
*Cis*-acting element analysis of m6A-related gene promoters from NL895. (**A**) Writers; (**B**) Erasers; (**C**) Readers.

To explore the potential regulatory functions of m6A-related genes in NL895, we identified *cis*-acting elements within the 2000 bp upstream of their transcription start sites. Four types of *cis*-elements were analyzed: plant development and growth, phytohormone response, light responsiveness, and stress responses (Figure 5). Nearly all m6A-related gene promoters contained at least one *cis*-acting element associated with plant development and growth, suggesting a role in development and growth processes in NL895. In addition, several *cis*-elements related to phytohormone responses, particularly ABRE and ERE, were abundant in the promoters of m6A-related genes, indicating a potential involvement in hormone regulation. Moreover, *cis*-elements associated with light responsiveness, such as Box-4 and G-box, were abundant in the promoters of these genes, suggesting involvement in light signaling. Interestingly, the G-box was also associated with jasmonic acid (JA) response, and promoters of m6A-related genes contained multiple G-box elements. Furthermore, the TGACG-motif and TGA-box, both linked to salicylic acid (SA), were found in many promoters, suggesting that these genes may be regulated by JA and SA. Additionally, all m6A-related gene promoters contained stress-related *cis*-elements, implying their involvement in stress responses. Notably, the promoters of writers contained more stress-related elements than those of erasers and readers, suggesting that writers may play a more significant role in stress responses.

### 3.7. Gene Expression Pattern Analysis of m6A-Related Genes

**Figure 6:**
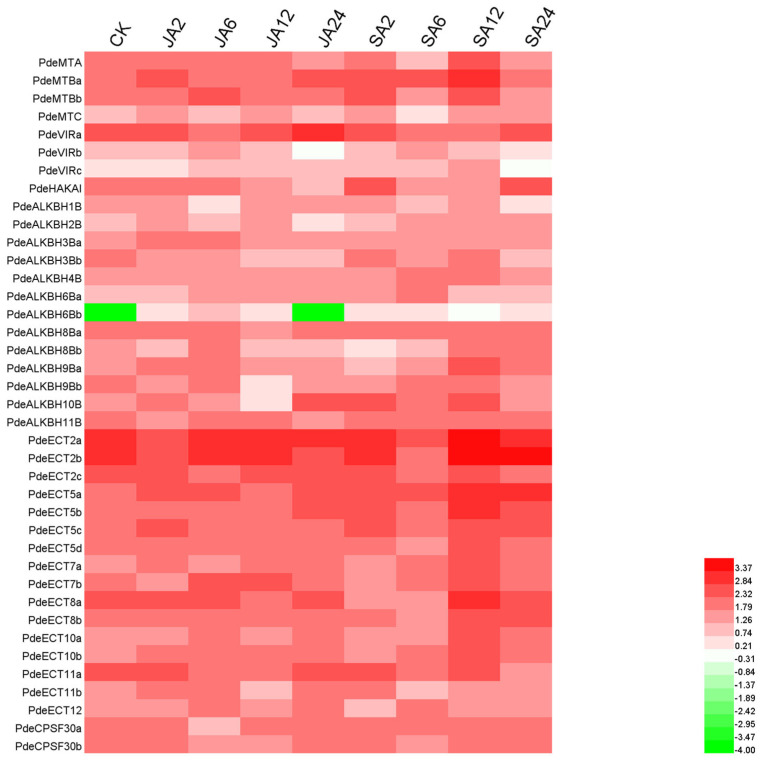
Expression of m6A-related genes after treatment with JA/SA for 0, 2, 6, 12, and 24 h. Red represents high expression, while green indicates low expression.

Given the *cis*-acting element analysis suggesting that m6A-related genes may be regulated by JA and SA, we examined the expression patterns of these genes under JA and SA treatment (Figure 6). The expression of some genes, such as *PdeMTBa* and *PdeALKBH6Ba*, was up-regulated after both JA and SA treatments. In contrast, the expression of other genes, such as *PdeALKBH8Ba*, *PdeALKBH11B*, and *PdeCPSF30b*, was down-regulated after JA and SA treatments. Interestingly, the promoter of *PdeECT2a* contains two TCA-elements, and its expression peaked 12 h after SA treatment. The expression of *PdeECT5d* continued to increase after JA treatment, which may be linked to five B-box elements in its promoter. The expression level of *PdeECT5a* increased more than twofold across all four time points after SA treatment, potentially due to the presence of three TGACG-elements in its promoter. These results suggest that *cis*-acting elements in the promoters of m6A-related genes play a crucial role in regulating gene expression in response to JA and SA.

### 3.8. Cadmium Stress Response of m6A Writers

**Figure 7:**
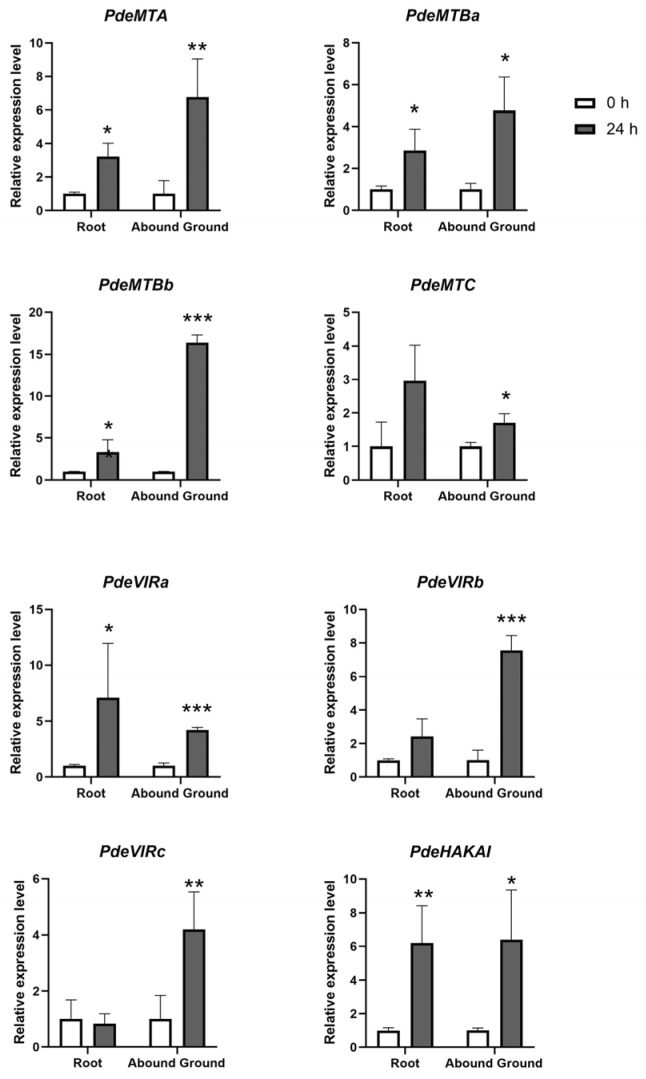
Relative expression levels of writers after 24 h cadmium treatment. White boxes indicate the 0 h cadmium treatment, and black boxes represent the 24 h cadmium treatment. Asterisks indicate significance levels (* *p* < 0.05, ** *p* < 0.01, *** *p* < 0.001).

Given the higher abundance of stress-related *cis*-elements in the promoters of writers compared to erasers and readers, we analyzed the expression levels of m6A writers under cadmium (Cd) stress. As shown in Figure 7, the expression of all writers in NL895, except *PdeVIRc*, were up-regulated after 24 h of cadmium treatment, both in the aerial and root tissues. Interestingly, after 24 h of cadmium stress, the expression levels of *PdeMTC* and *PdeVIRa* were higher in the aerial part than in the root, while the expression levels of other writers were higher in the root. These results suggest that writers play a significant role in the cadmium stress response in NL895.

## 4. Discussion

### 4.1. Characteristics and Functional Evolution of m6A-Related Genes in NL895

m6A, an essential post-transcriptional epigenetic regulatory mechanism in eukaryotes, plays a critical role in various biological processes, including plant growth, development, stress response, and signal transduction. In this study, we systematically analyzed the m6A-related gene family (comprising writers, erasers, and readers) in *Populus* NL895, providing insights into gene characteristics, structural diversity, phylogenetic relationships, and the regulatory potential of *cis*-acting elements. Our findings offer a novel theoretical foundation for understanding the epigenetic regulatory networks in woody plants.

### 4.2. Genome-Wide Identification of m6A-Related Genes

We identified 39 m6A-related genes in *Populus* NL895, with the following distributions: 20.5% methyltransferases (writers), 33.3% demethylases (erasers), and 46.2% recognition proteins (readers) (Figure 1). Notably, NL895 had a higher number of writers and readers compared to *Arabidopsis* and *Oryza sativa*, suggesting an expansion of these gene families in *Populus*. Gene duplication, often driven by whole-genome duplication (WGD) events, likely contributed to this expansion [44,45]. Our analysis revealed that all duplicated m6A-related genes in NL895 originated from WGD events. Specifically, two copies of the *MTB* gene (*PdeMTBa* and *PdeMTBb*) were identified on chromosomes 12 and 15, supporting the role of WGD in gene family expansion. The duplicated gene pairs predominantly underwent purifying selection, indicating that their evolutionary fate may involve subfunctionalization rather than neofunctionalization [46,47,48].

### 4.3. Chromosome Localization and Evolutionary Conservation

The m6A-related genes in NL895 were unevenly distributed across 16 chromosomes, with six m6A genes (including 1 writer, 1 eraser, and 4 readers) clustered on chromosome 1. This suggests that this chromosomal region could be involved in the regulation of epigenetic “hot spots” in *Populus*. Phylogenetic analysis revealed that the gene family members of writers and erasers in NL895 showed high evolutionary conservation with dicotyledonous plants like *Arabidopsis,* compared to monocotyledonous plants like *Oryza sativa* [49,50]. In contrast, readers exhibited high conservation across both monocots and dicots, and were divided into two distinct clades: YTHDF and YTHDC (Figure 2C). Additionally, gene structure analysis indicated that writers typically had more exons than erasers and readers, which may reflect functional complexity and regulatory hierarchy differentiation among the different m6A categories.

### 4.4. Cis-Acting Elements and Their Role in Gene Expression Regulation

m6A plays a pivotal role in regulating plant responses to environmental stimuli. Our analysis of the promoters of m6A-related genes in NL895 identified abundant light-responsive *cis*-elements, such as G-box and Box-4 (Figure 5). These findings suggest that m6A modification may play a significant role in mediating light signal transduction, potentially influencing processes such as photomorphogenesis and circadian rhythm regulation. For example, cryptochrome 2 has been shown to recruit the m6A methyltransferase complex (MTA/MTB/FIP37) through liquid–liquid phase separation upon photoexcitation to regulate gene expression [51]. Furthermore, GhALKBH5, a demethylase in cotton, regulates photoperiodic flowering genes, influencing the plant’s photoperiodic sensitivity [52]. The dynamic regulation of m6A levels in response to light conditions could therefore serve as a mechanism to optimize gene expression for improved photosynthetic efficiency and energy utilization.

In addition to light response, the promoters of m6A-related genes were enriched with hormone-responsive *cis*-elements, highlighting their involvement in hormone signaling pathways. Recent studies have revealed a feedback regulatory mechanism between m6A and auxin biosynthesis, which plays a critical role in forming local auxin maxima during male meiosis in rice anthers [53]. In *Arabidopsis*, phase separation mediates m6A modification of ABA receptor mRNA, negatively regulating ABA perception [54]. In NL895, the dynamic expression changes in m6A-related genes after jasmonic acid (JA) and salicylic acid (SA) treatment further suggest that these hormones might regulate gene expression via *cis*-acting elements. For instance, the promoter of *PdeECT2a* contained the most TGA motifs, and *PdeECT5a* contained the most TGACG motifs in the readers. Both genes showed the highest expression after 12 h of SA treatment. Interestingly, the expression of some genes exhibited opposite trends under JA and SA treatment, indicating that these genes may have different regulatory roles in response to these hormones. Notably, *PdeECT2a* and *AtECT1* (an m6A reader in *Arabidopsis*) were found in the same phylogenetic clade, suggesting a potential similarity in their roles in SA response [55]. Furthermore, certain genes exhibited similar expression patterns under JA and SA treatments, such as *PdeMTAa*, *PdeALKBH8Ba*, *PdeALKBH11B,* and *PdeCPSF30b*, indicating that these genes may contribute to potential synergies between the JA/SA pathways.

### 4.5. m6A in Plant Stress Response

m6A modification plays a crucial role in plant stress responses [22,56,57,58,59,60,61]. For example, in sweet sorghum, salt stress increased m6A modification and the mRNA stability of salt resistance-related genes, enhancing salt tolerance [22]. *MTA* was also implicated in promoting drought resistance in *Populus* by regulating trichoid and root development [57]. *PagFIP37* was shown to mediate the methylation of N6-methyladenosine mRNA, positively regulating the response of poplar to salt stress [58]. *PagALKBH9B* and *PagALKBH10B* were shown to regulate the salt stress response in *Populus* [59]. *MdVIR1* and *MdVIR2* enhanced apple resistance to *Alternaria alternata* by mediating m6A methylation of *MdWRKY79* and *MdNLR16* mRNAs, thereby stabilizing transcripts and improving translation efficiency in response to sorbitol and pathogen stress [60]. Additionally, *O. sativa* studies showed that cadmium stress alters m6A modifications on root transcripts, potentially disrupting root development [61]. In NL895, cadmium stress led to the up-regulation of all writers in both root and aboveground parts, which may be linked to the abundant stress-related *cis*-acting elements in their promoters. These results suggest that m6A writers play an important role in responding to cadmium stress in *Populus*.

## 5. Conclusions

In this study, we identified 8 writers, 13 erasers, and 18 readers in poplar NL895. Whole-genome duplication (WGD) events were the key drivers in the expansion of the writer and reader gene families. The *Ka*/*Ks* ratios of all duplicated genes were less than 1, indicating purifying selection and potential subfunctionalization. Phylogenetic analysis revealed that writers, erasers, and readers clustered into six, four, and two distinct groups, respectively, with conserved features such as domains and motifs shared within each group. *Cis*-acting element analysis indicated that promoter elements likely regulate gene expression in response to hormonal signals, particularly jasmonic acid (JA) and salicylic acid (SA). Additionally, the qPCR data suggest that writers play a critical role in the response to cadmium stress. Future research should focus on validating the regulatory roles of m6A-related genes through functional studies and further investigating the interplay between m6A modification and other epigenetic mechanisms. Understanding the molecular basis of m6A-mediated regulation could offer valuable insights into enhancing stress tolerance and productivity in poplar and other economically important tree species.

## Figures and Tables

**Table 1:** Ka/*Ks* ratios and duplication types of m6A-related gene pairs.

Duplicated Gene Pairs	*Ka*	*Ks*	*Ka*/*Ks*	Duplication Type	Selection
*PdeMTBa*/*PdeMTBb*	0.066449	0.243759	0.272603	WGD	Purifying
*PdeALKBH6Ba*/*PdeALKBH6Bb*	0.295386	0.615411	0.479981	WGD	Purifying
*PdeALKBH8Ba*/*PdeALKBH8Bb*	0.078067	0.265596	0.293931	WGD	Purifying
*PdeALKBH9Ba*/*PdeALKBH9Bb*	0.102159	0.282231	0.36197	WGD	Purifying
*PdeECT2a*/*PdeECT2b*	0.119733	0.387067	0.309334	WGD	Purifying
*PdeECT2b*/*PdeECT2c*	0.354481	2.112958	0.167765	WGD	Purifying
*PdeECT2c*/*PdeECT2a*	0.290612	1.626234	0.178703	WGD	Purifying
*PdeECT5c*/*PdeECT5b*	0	0.022474	0	WGD	Purifying
*PdeECT7a*/*PdeECT7b*	0.098606	0.249897	0.394586	WGD	Purifying
*PdeECT10a*/*PdeECT10b*	0.085928	0.311314	0.276016	WGD	Purifying
*PdeECT11a*/*PdeECT11b*	0.073604	0.221573	0.33219	WGD	Purifying
*PdeCPSF30a*/*PdeCPSF30b*	0.053137	0.309897	0.171467	WGD	Purifying

## Data Availability

The raw RNA-seq data came from the NCBI database (https://www.ncbi.nlm.nih.gov/bioproject/PRJNA511770, accessed on 29 June 2025), with the accession number (SRR8371728-SRR8371754).

## Supplementary Materials

The following supporting information can be downloaded at https://www.mdpi.com/article/10.3390/plants14132017/s1, Figure S1: Motif analysis of m6A-related genes. (A) Motif location and logo of writers. (B) Motif location and logo of erasers. (C) Motif location and logo of readers; Table S1: The primer used in qPCR; Table S2: Gene ID, Gene name and amino acid sequences of m6A-related gene family from NL895; Table S3: The five duplicated types of m6A-related genes from NL895; Table S4: The predicated protein information of m6A-related genes from NL895.

## List of Abbreviations

*Populus deltoids* × *P. euramericana*: NL895; N6-methyladesine: m6A; m6A methyltransferases: writers; m6A methyltransferases: erasers; m6A recognition enzymes: readers; Molecular weight: MW; Grand average of hydropathicity: GRAVY; Instability index and isoelectric point: pI; Real-time qPCR: RT-qPCR; Whole-genome duplication: WGD; Dispersed duplication: DSD; Tandem duplication: TD; Proximal duplication: PD; Transposed duplication: TRD; *Arabidopsis thaliana*: *A. thaliana*; *Oryza sativa*: *O. sativa*; *Populus trichocarpa*: *P. trichocarpa*; Amino acids: aa; Jasmonic acid: JA; Salicylic acid: SA.
